# Development and evaluation of a virtual reality basic life support for undergraduate students in Thailand: a project by Mae Fah Luang University (MFU BLiS VR)

**DOI:** 10.1186/s12909-023-04764-6

**Published:** 2023-10-19

**Authors:** Boonyapat Shatpattananunt, Wongchan Petpichetchian, Soifah Pinsuwan, Tanyawut Chaloempong, Sineenat Waraphok, Charoenchai Wongwatkit

**Affiliations:** 1 Nursing Innovation Research and Resource Unit, School of Nursing, Mae Fah Luang University, Chiang Rai, Thailand; 2 College of Advanced Practice Nurse and Midwife, Thailand Nursing and Midwifery Council, Nonthaburi, Thailand; 3 School of Nursing, Mae Fah Luang University, Chiang Rai, Thailand; 4 Learning Innovation Institute, Mae Fah Luang University, Chiang Rai, Thailand; 5 Faculty of Nursing, Chulalongkorn University, Bangkok, Thailand

**Keywords:** Immersive training, Virtual reality, Learning simulation, Basic life support, Research and Development

## Abstract

**Background:**

In traditional basic life support training for university students or the public, trainees practice simulations only once or twice during the course, potentially limiting their competence. In contrast, virtual reality allows trainees to independently study and practice as often as needed, enhancing their skills. This research and development project aimed to develop and evaluate a novel learning device, virtual reality basic life support for undergraduate students of Mae Fah Luang University (MFU BLiS VR).

**Methods:**

This study employed a two-group, pre- and post-test design, involving seventy students (*n* = 35 in each group) from Mae Fah Luang University, Thailand. Data were collected from March 2022 to January 2023. The experimental group received the MFU BLiS VR, in addition to traditional teaching, while the control group received only traditional teaching. Data analysis employed descriptive statistics, Chi-square, Mann-Whitney U test, and Wilcoxon signed ranks test.

**Results:**

“MFU BLiS VR” provided a learning experience in out-of-hospital basic life support for adult patients in four scenarios: (1) a person who was not breathing but had a pulse; (2) a person who was not breathing, had no pulse, and required defibrillation; (3) a person who was not breathing, had no pulse, and did not require defibrillation; and (4) a person with normal breathing and pulse but was unconscious. Each scenario was presented sequentially from scenario one to scenario four. The scenarios encompassed common and complex situations requiring prompt and effective bystander responses to save lives. The results revealed that the experimental group had a significantly shorter no-flow time compared to the control group (Z = -5.02, *p* < .001) and achieved significantly higher knowledge scores than the control group (Z = -3.39, *p* < .01) as well as superior practical skills (Z = -7.26, *p* < .001). Both groups reported the highest satisfaction levels in all aspects, with no significant differences.

**Conclusion:**

MFU BLiS VR is an effective training approach for teaching and learning basic life support and the use of an automated electronic defibrillator. It captures students’ attention and enhances their understanding of these essential life support skills, which are crucial for everyone.

**Supplementary Information:**

The online version contains supplementary material available at 10.1186/s12909-023-04764-6.

## Background

 Out-of-hospital cardiac arrest (OHCA) is a medical emergency and a significant public health concern [1]. It remains one of the leading causes of death worldwide [1–5]. In Europe, the estimated incidence of OHCA ranges from 67 to 170 cases per 100,000 population per year [6]. In the United States, sudden cardiac deaths are estimated to account for approximately 380,000 cases per year [7]. A systematic review and meta-analysis study [1], which analyzed 141 studies published globally between 1976 and 2019, reported a pooled incidence of return of spontaneous circulation (ROSC) among OHCA patients at 29.7% (95% CI 27.6–31.7%) with significant heterogeneity across the studies. Furthermore, this study revealed that when compared to studies from other continents (Oceania, Europe, and North America), Asia had the lowest incidence of prehospital ROSC (22.1%) and survival to admission rate (15.6%) among OHCA patients receiving cardiopulmonary resuscitation (CPR). A recent systematic review and meta-analysis study based on 49 studies in China reported the survival outcomes of OHCA patients receiving out-of-hospital CPR were far below the global average [5]. They found that the pooled incidence of ROSC and survival to admission rate in OHCA patients in China were only 9% and 5%, respectively.

Several factors may influence the survival rate of OHCA, including age, underlying diseases, the causes of cardiac arrest, response time, and treatment period [8]. Key factors associated with ROSC among OHCA patients include the public location and time of arrest, an initial shockable rhythm, early recognition by bystanders, prompt initiation of CPR, and the use of an automated external defibrillator (AED) [9]. The presence of a bystander witness and the administration of bystander CPR have been identified as factors influencing the incidence of survival to hospital discharge [1, 5] Moreover, a study conducted in New Taipei City, Taiwan highlighted the specific factors in achieving ROSC, including maintaining a chest compression fraction (CCF) greater than 0.8 and minimizing chest compression interruption (less than 3 times) [10]. Given the significance of these factors, providing training in basic life support (BLS) to bystanders in the general public is crucial for enhancing the likelihood of positive outcomes in cases of OHCA.

BLS for adults experiencing OHCA follows the Chain of Survival, which consists of six key steps: (1) early recognition and prevention, (2) activation of emergency response, (3) high-quality CPR, (4) defibrillation, (5) post-cardiac arrest care, and (6) recovery. Effective patient care relies on community engagement and responsiveness. Once an incident is recognized, individuals should call 1669, Thailand’s local number for emergency medical service (EMS), perform CPR, and use an AED while waiting for the EMS team to arrive [11]. Traditional face-to-face CPR training involves teaching individuals how to perform CPR on a manikin under the guidance of a certified instructor [12]. During this training, trainees learn to check for responsiveness, call for help, assess breathing, administer chest compressions, provide rescue breathing, and use an AED. While this training method has increased the number of bystander CPRs, it has limitations in terms of realistic performance and immersion. These limitations became evident during the COVID-19 pandemic when face-to-face training was not readily available [13].

Due to the challenges posed by COVID-19 in providing a safe clinical practice environment, virtual reality (VR) has emerged as an alternative to hands-on training [14]. VR is a computer-based multimedia environment that allows users to interact with a computer-generated world, providing a realistic sensory experience despite its existence in the virtual realm. [15, 16]. Immersive environments assist students in learning complex content and in developing creative, technical, and problem-solving skills. This form of interactive learning has evolved from active learning and engagement, allowing students to explore VR to find innovative solutions to real-world problems [17].

Previous studies have primarily focused on using VR for CPR scenarios involving individuals, and one of the four scenarios in BLS training. These studies have consistently demonstrated that VR technology is an effective tool for BLS training. This effectiveness can be attributed to its capacity to provide immersive, multisensory, interactive 3D multimedia simulations and realistic learning environments [12, 17–20]. Moreover, VR allows BLS experiences to be brought into the classroom, creating a valuable learning opportunity. Notably, students who utilized VR in group learning settings demonstrated significantly better BLS performance than those who studied through other traditional learning methods [13, 21, 22]. These findings underscore the importance of incorporating VR into BLS training. However, it is worth noting that certain limitations of VR technology may hinder the practice of techniques, such as CCF and AED.

Recognizing the importance of effective BLS training, the quality of CPR, the potential of VR to enhance a student’s overall learning experience, and the need for accessible and repeatable learning anytime and anywhere, this study aimed to develop and evaluate a training course, namely virtual reality basic life support for undergraduate students of Mae Fah Luang University (MFU BLiS VR). The rationale for focusing on university students stems from their status as valuable human resources, both physically and mentally capable, compared to other age groups. Providing this group with training equips them to potentially save lives when the need arises. We hypothesized that the experimental group receiving the MFU BLiS VR would demonstrate shorter no-flow time (primary outcome), higher levels of BLS knowledge, BLS practical skills (secondary outcomes), and greater satisfaction (tertiary outcome) compared to the control group.

## Methods

### Design

A research and development (R&D) project was conducted. The effectiveness of the developed MFU BLiS VR training was evaluated using a two-group, pre- and post-test design. The primary source of content for the training was the basic life support (BLS) guidelines for adults experiencing out-of-hospital cardiac arrest (OHCA) developed by the American Heart Association (AHA), [23]. The research and development methodology outlined by Borg and Gall [24] guided the process conducted in this study.

### Sample and setting

In this project, three participants were involved in preliminary field testing (R1), seven participants were selected to participate in the main field testing (R2), and 70 participants were chosen to assess the effectiveness of the MFU BLiS VR in operational field testing (R3). The sample size for R3 was determined by using G*Power software version 3.1 for a one-tailed hypothesis test with an independent t-test. The desired power was set at 0.95, the effect size at 0.80, and a significance level at 0.05. Consequently, the sample size was calculated to be 35 participants per group [25]. Additionally, a matched pairs design was employed based on personal data, including gender, age, education level, and prior training experience in adult OHCA BLS. After obtaining ethical approval, the principal investigator (PI) presented the research project’s banner to the Line groups of student club presidents. Students who met the inclusion criteria were invited to participate voluntarily. The selection process involved categorizing all participants based on their personal information to ensure that each group had a similar composition. A simple random sampling method without replacement was employed to assign participants to either the experimental group or the control group. Notably, no participants withdrew or were missing from this study.

The participants were undergraduate students recruited through purposive sampling based on the following inclusion criteria: (1) enrollment at Mae Fah Luang University; (2) age 18 years old or above; (3) willingness to enroll in an extracurricular BLS training course arranged for this study; (4) proficiency in the Thai language; and (5) no prior BLS training within the last year, as a previous study indicated a statistically significant decrease in BLS knowledge after a year of training at the 0.01 level [26]. Exclusion criteria were applied to individuals with health conditions preventing their participation in this study (e.g., visual impairment) and those who did not adhere to the study’s instructions.

### Research instruments

Research instruments comprised of two parts: data collection instruments and the intervention course.

*The Data collection instruments* consisted of four instruments, all of which underwent content validation by three experts: an emergency physician, a paramedic nurse, and an education technology professor. The index of item-objective congruence (IOC) was assessed by the experts and ranged from 0.66 to 1. Reliability was pilot-tested with 18 participants who met the same inclusion criteria as those in the study.

*The Demographic Questionnaire* comprised of four closed-ended questions related to gender, age, education level, and training experience in adult OHCA basic life support.

*The BLS Knowledge Questionnaire (BKQ)* consisting of 10 items, was developed by Wittayachamnamkul et al. [27]. Each correct response was scored as 1, while an incorrect response received a score of 0. A higher score indicated a higher knowledge of BLS for adult OHCA. Test-retest reliability, conducted one week apart, yielded an intraclass correlation coefficient (ICC) for the BKQ of 0.76.

*The BLS Skill Checklist (BSC)* consisted of 25 items developed based on the literature, particularly the American Heart Association’s guidelines [23]. Each item was rated on a 0–2 point scale (0 = not practice, 1 = incomplete practice, 2 = complete practice). The participants’ practical skills were assessed by the research assistants. To test the equivalence (inter-rater reliability) of the BSC, two members of the research team independently observed the pilot participants, resulting in a Cohen’s Kappa reliability coefficient of 0.63.

*The Satisfaction Scale* developed by the researchers consisted of 6 items designed to assess a participant’s perceived satisfaction with the BLS training course. Each item featured a 5-point Likert scale for satisfaction responses (5 = very satisfied, 4 = satisfied, 3 = neither, 2 = dissatisfied, and 1 = very dissatisfied). The total score ranged from 6 to 30 with the higher scores indicating higher satisfaction with the BLS training course. The Cronbach’s alpha coefficient for the internal consistency reliability of this scale was 0.93.

### Intervention

*Essential equipment for the MFU BLiS VR training course for adult OHCA* includes VR hardware (virtual reality headset and VR remote controller), a pillow size 19 × 29 inches, and a half-body training manikin for CPR practice (Prestan professional training manikin: PP-AM-400 M-MS).

#### Virtual reality module development stages

The MFU BLiS VR was developed step by step following the research and development methodology outlined by Borg and Gall [24]. The procedures were described as follows: (1) research and information collection, (2) planning, (3) developing a preliminary product, (4) preliminary field testing, (5) revising the main product, (6) main field testing, (7) revising the operational product, (8) operational field testing, (9) revising the final product, and (10) disseminating and implementing. For this present study, eight steps were employed as follows.

1. Developing a preliminary form of an initial draft of the MFU BLiS VR (D1). The AHA 2020 BLS’s guidelines were chosen as the main contents of the four included scenarios: (1) a person who was not breathing but had a pulse, (2) a person who was not breathing, had no pulse, and required defibrillation, (3) a person who was not breathing, had no pulse, and did not require defibrillation, and (4) a person with normal breathing and pulse but was unconscious. In each step of the scenario, participants are required to select the appropriate equipment (such as a defibrillator in the second scenario). Failure to make all the necessary choices prevents the game from progressing, resulting in the participant spending a significant amount of time on the task. This can impact the total time taken and may reflect the participant’s ability to recall the correct steps. The fourth scenario represents a common situation where a person from scenarios 1, 2, and 3 has received help and achieved ROSC.

The selected location was a building at Mae Fah Luang University. Each scene featured a Thai voiceover and included subtitles in both English and Thai. The researchers initially drafted the MFU BLiS VR using Unity, a versatile multi-platform game engine for developing interactive 3D content. It was accessible on smartphones with at least Android 8 + versions and with certain parts and features requiring a password for access.

The content of the initial draft of the MFU  BLiS VR was validated by a group of three experts, similar to those who examined the data collection instruments. The experts recommended adjusting the Thai language to resemble English while conveying the same meanings, displaying a time bar on the screen during VR play, and implementing a data security system accessible only through registered email addresses.

2. Preliminary field testing (R1) of the initial draft of the MFU BLiS VR. This step involved three participants who provided feedback on the product’s interactivity and quality. The participants mentioned that the head-up display (HUD) system during VR gameplay was too close to the eyes, potentially causing distraction and blurriness in some cases. They also noted that the user interface (UI) was incomplete, thus making it challenging to comprehend the on-screen events.
3. Revising the main product (D2). The researchers made improvements to the initial draft of MFU BLiS VR based on the feedback from R1. These enhancements included expanding the field of view for a more precise view, repositioning the HUD to be further from the player’s line of sight, and incorporating descriptions and images to provide better guidance for participants to understand the scenario on the screen. With these modifications, the second draft of the MFU BLiS VR was prepared for the next step.
4. Main field testing (R2). The second draft of the MFU BLiS VR was evaluated with seven participants. The researchers employed a qualitative method to collect data and gather insights from the participants. Feedback from the participants indicated that they found some scenes in the VR experience to be lackluster due to excessively lengthy text. They also expressed uncertainty about the exact rate and number of compressions while assisting in the VR scenario. The participants mentioned that the hitboxes on the manikin were too small and sometimes unreachable from certain positions. Additionally, some scenes had multiple stages that lacked coherence, thus diminishing the overall enjoyment of the VR experience.
5. Revising operational product (D3). In this step, the researchers made adjustments to the second draft of the MFU BLiS VR using data gathered in step four. These included making the VR text concise and direct, enhancing the clarity of the hitbox size and colors, and streamlining some steps in certain scenes to improve the overall flow of the MFU BLiS VR. As a result, a third draft of MFU BLiS VR was developed as a validated operational model design.
6. Operational field testing (R3). This step involved 70 participants in a two-group, pre- and post-test research design aimed at assessing differences in learning outcomes between two groups. The experimental group utilized the third draft of the MFU BLiS VR for learning, while the control group received traditional teaching.
7. Revising the final product (D4). In this step, the refinement led to the creation of a final draft of the MFU BLiS VR (see Supplement file 1). This final draft incorporated numerous product enhancement recommendations from step six.
8. Disseminating and implementing. During this step, the MFU BLiS VR training course for adult OHCA was integrated as a component of the emergency, trauma, and disaster nursing course for third-year nursing students, totaling 120 students. After completing the MFU BLiS VR for adult OHCA training course, the participants received a certificate from the School of Nursing at Mae Fah Luang University. A notable feature of this innovation was its compatibility with a wide range of Android smartphones, virtual reality headsets, and motion controllers, all of which were available for less than 30 USD. Students had the option to purchase or borrow these devices from the Nursing Learning and Resource Center. A guiding principle behind the development of this innovation was its accessibility to everyone and it was widely disseminated.

### Data collection

This study was conducted from March 2022 to January 2023. Three Thai nursing instructors assisted as research assistants (RAs). They underwent training in BLS and were proficient in handling the four research instruments. The researchers obtained informed consent from the participants and scheduled their appointments. One RA administered the baseline questionnaires, while the other two observed the participants during the practice examination, immediately following the face-to-face BLS training session in a classroom. To lessen measurement bias, a single-blind technique was employed, with the research assistants being blinded to group assignments.

**Fig. 1:**
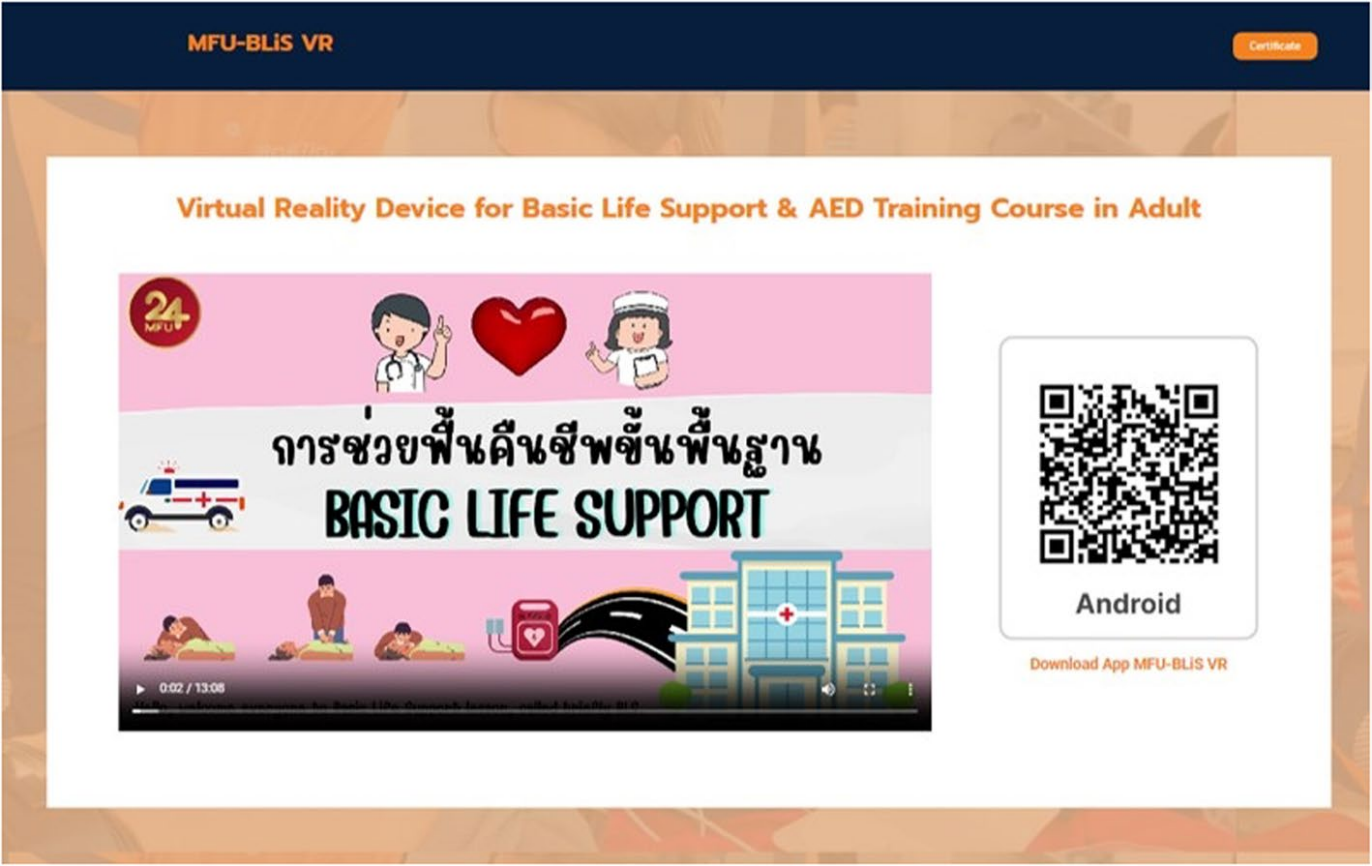
Display the webpage of MFU BLiS VR and a BLS instructional video clip

**Fig. 2:**
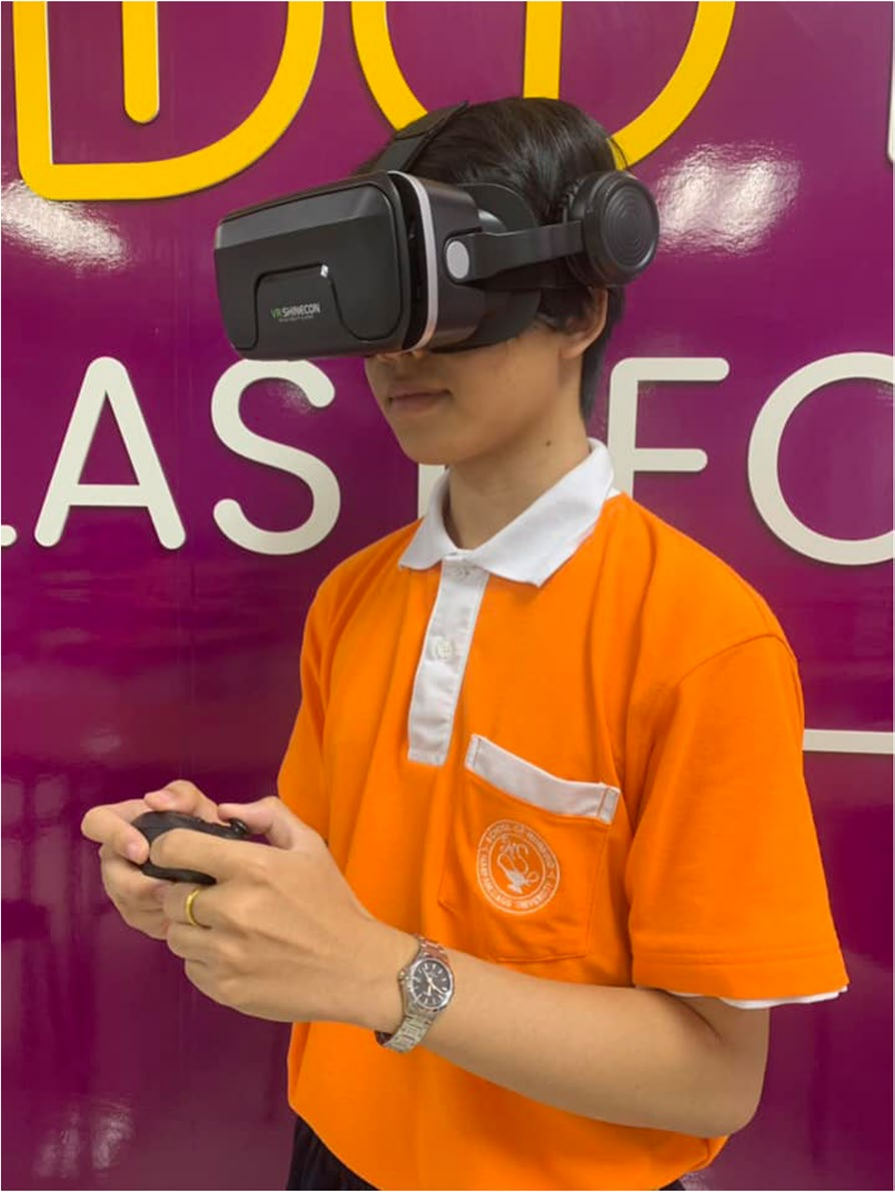
Display of a VR headset and VR remote controller for individual experience with MFU BLiS VR

**Fig. 3:**
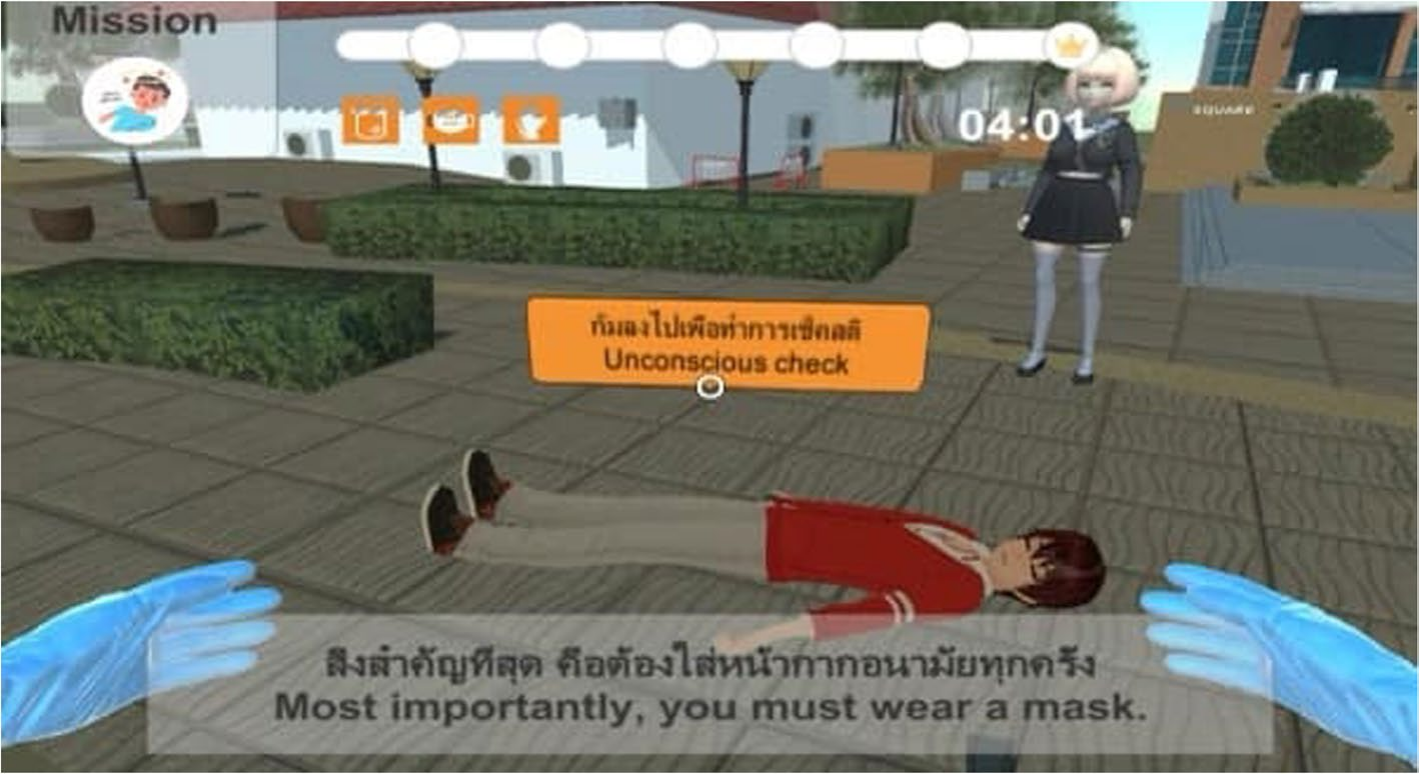
Participants viewed a MFU BLiS VR training with Thai voiceover and bilingual subtitles through a VR headset

**Fig. 4:**
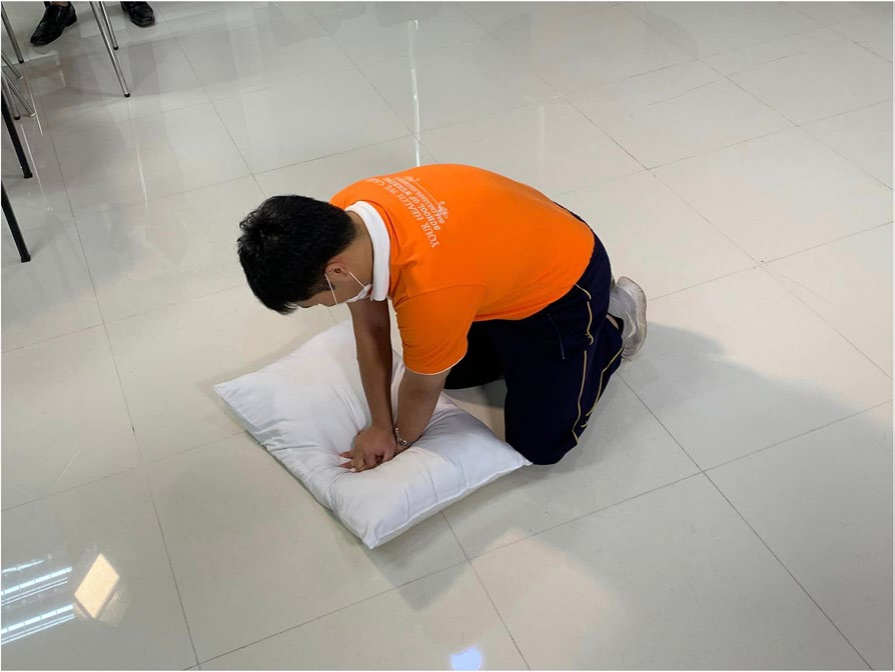
Practice BLS with a pillow, focusing on the participant’s position, rhythm, and steps of CPR

**Fig. 5:**
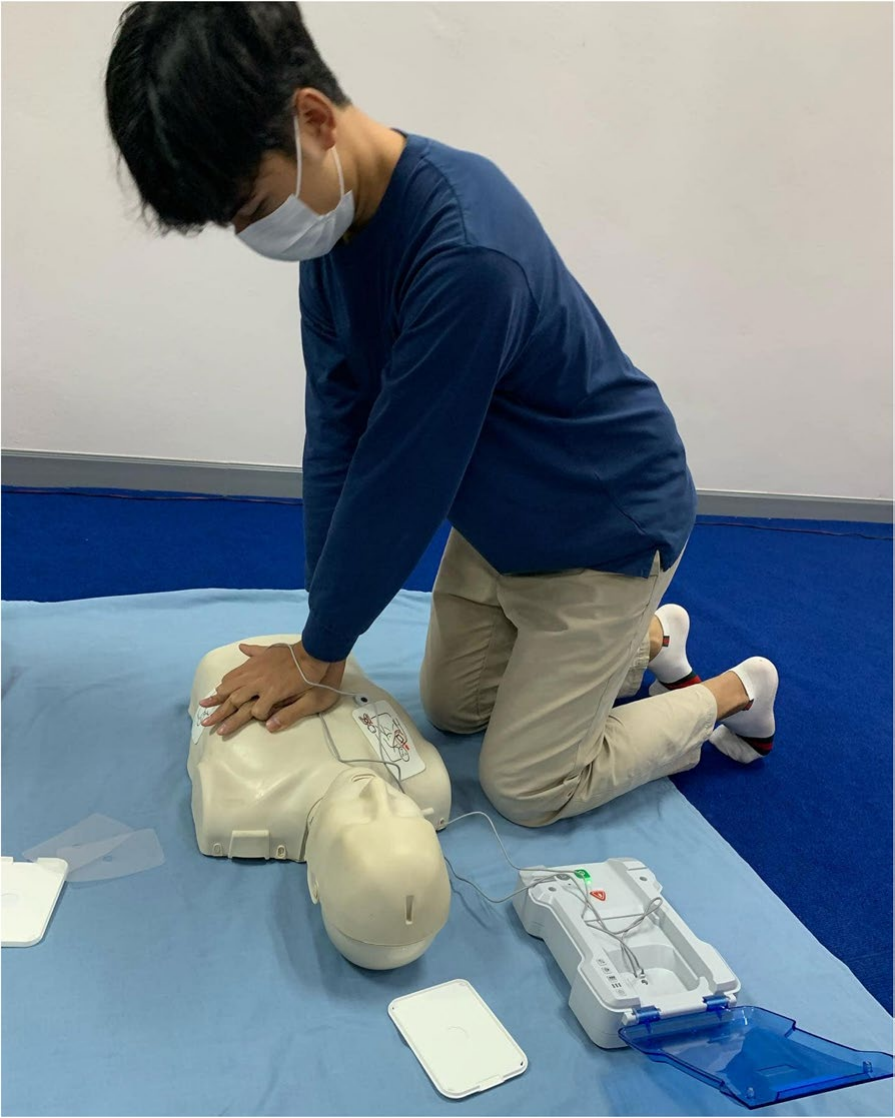
Practice BLS and use of an AED with a half-body training manikin in the classroom under the RA’s supervision

 The PI designed the MFU BLiS VR training course for adult OHCA for the experimental group, consisting of six phases: (1) a pre-test assessing BLS knowledge using the BKQ and collecting demographic data; (2) watching a BLS instructional video clip (Fig. 1); (3) individual experience with MFU BLiS VR (Figs. 2 and 3); (4) CPR practice using a pillow as a half-body training manikin (Fig. 4); (5) classroom-based face-to-face BLS training with a research assistant, assessing practical skills using the BSC and timing the no-flow time (Fig. 5); and (6) a post-test evaluating BLS knowledge using BKQ and assessing perceived satisfaction with the BLS training course using the Satisfaction Scale. It is worth noting that, the online materials and the MFU BLiS VR allow participants to engage in self-directed learning, providing flexibility in terms of when and where they choose to study. The control group underwent the same phases with the exception of phase three (3). To uphold the ethical principles of beneficence and justice, the control group was granted access to the MFU BLiS VR after the study concluded.

### Data analysis

Descriptive statistics were employed to analyze the participants’ demographics. The Chi-square test was used to examine differences in demographic data between the two groups. As the assumption of normal distribution for no-flow time (minutes), as well as the scores of BLS knowledge, practical skills, and perceived satisfaction with the BLS training course, was not met, the Mann-Whitney U test was used to compare the mean ranks of these variables between the two groups. The Wilcoxon signed ranks test was used to compare the mean ranks of the pre-test and post-test regarding BLS knowledge within each group. A significance level was set at 0.05.

## Results

### Homogeneity of the subject

There were 70 participants in this study with no significant differences in demographic characteristics between the experimental and control groups in terms of gender, age, education level, and training experience in adult OHCA BLS. Most participants were female (68.6%) with ages ranging from 18 to 22 years (Mdn = 20, IQR = 2). Nearly half were in their second year of education (32/45.71%). The majority of the participants had no prior training experience in BLS (60/85.71%) (Table 1).

**Table 1:** Demographic characteristics of participants

Personal demographic	Experimental group (*n* = 35)		Control group (*n* = 35)		χ^2^	*p*
	n	%	n	%		
Gender					0.00	1.00
Female	24	68.6	24	68.6		
Male	11	31.4	11	31.4		
Age (years)^a^	Mdn = 20.0		Mdn = 20		0.07	0.97
	IQR = 2		IQR = 2			
	Min = 18		Min = 18			
	Max = 22		Max = 22			
Education level					0.00	1.00
First-year	6	17.1	6	17.1		
Second-year	16	45.8	16	45.8		
Third-year	13	37.1	13	37.1		
Prior training experience in adult OHCA BLS					0.00	1.00
No	30	85.7	30	85.7		
Yes	5	14.3	5	14.3		
Trained within 1 year	2	40.0	2	40.0		
2 years	3	60.0	3	60.0		

^a^ Mann-Whiney U Test

### Effectiveness of MFU BLiS VR

Descriptions of each outcome variable are displayed in Tables 2 and 3. The pre-test and post-test mean ranks on BLS knowledge of both groups were significantly different, in which the post-test scores on BLS knowledge were higher than the pre-test scores, as shown in Table 2. Comparisons between the two groups with regard to no-flow time, BLS knowledge, practical skills, and perceived satisfaction with the BLS training course, as presented in Table 4, indicated that the mean rank of no-flow time in the experimental group was significantly shorter than that of the control group (Z = -5.02, *p* < .001). Moreover, the mean ranks of BLS knowledge and BLS practical skills in the experimental group were significantly higher than those of the control group (Z = -3.39, *p* < .01 and Z = -7.26, *p* < .001, respectively). In contrast, there was no significant difference in perceived satisfaction between the two groups (Z = -0.31, *p* > .05).

**Table 2:** Descriptions and comparisons of the pre-test and post-test BLS knowledge scores of the experimental and control groups using the Wilcoxon Signed Ranks Test

BLS Knowledge	Pre-test			Post-test			Mean Rank	Z	*p*
	n	Min-Max	Mdn (IQR)	n	Min-Max	Mdn (IQR)			
Experimental group	35	2–9	6 (2)		7–10	10 (1)		-5.18	0.000
Negative ranks	0						0		
Positive rank	35						18		
Ties	0								
Control group	35	2–10	5 (2)		6–10	9 (1)		-4.90	0.000
Negative ranks	0						0		
Positive rank	31						16		
Ties	4								

Negative ranks: post-test < pre-test; Positive ranks: post-test > pre-test: Ties: post-test = pre-test

*Min* Minimum score, *Max *Maximum score, *Mdn *Median, *IQR *Interquartile range

**Table 3:** Descriptions of no-flow time, BLS practical skills, and perceived satisfaction with the BLS training course of the experimental and control groups after completing the course

Variables	Experimental Group (*n* = 35)				Control Group (*n* = 35)			
	Min	Max	Mdn	IQR	Min	Max	Mdn	IQR
No-flow time (minute)	3	5	5	1	4	13	6	2
BLS practical skill	48	50	50	1	25	49	41	8
Perceived satisfaction	19	30	30	1	24	30	30	0

*Min* Minimum score, *Max *Maximum score, Mdn Median, *IQR *Interquartile range

**Table 4:** Comparison of no-flow time, the post-test BLS knowledge, BLS practical skills, and perceived satisfaction with the BLS training course between the experimental and control groups using the Mann-Whitney U test

Variable	Experimental Group	Control Group	Z	*p*
	Mean rank	Mean rank		
No-flow time	24.29	46.71	-5.02	0.000
BLS knowledge	43.34	27.66	-3.39	0.001
BLS practical skills	52.69	18.31	-7.26	0.000
Perceived satisfaction	34.94	36.06	-0.31	0.775

## Discussion

No-flow time in this study was defined as the total duration during which chest compressions were not performed on a cardiac arrest patient. Reducing no-flow time is essential in CPR because it helps maintain blood circulation and oxygen delivery to the brain and other vital organs. No-flow time or no-flow interval has been found to be inversely associated with favorable neurological outcomes [28]. Therefore, it is commonly used as a sensitive indicator of the quality of resuscitation [21, 22]. Our study demonstrated that the experimental group had significantly shorter no-flow time than the control group. This could be attributed to the fact that individuals with precise knowledge and memorization of the process tend to perform more accurately and quickly. This finding aligns with a randomized controlled trial conducted in Germany during the COVID-19 pandemic [21] to assess the effectiveness of VR BLS training. The researchers reported significantly lower no-flow time in the VR BLS training group compared to a control group that received web-based training. However, in contrast, a randomized controlled trial involving first-year medical students compared the no-flow time between the experimental group, which received a 35-min VR BLS training, and the control group, which received a traditional BLS course with a seminar. Surprisingly, this study found that the no-flow time was significantly lower in the control group than in the experimental group. They reported that it might be attributed to the participants in their study only using virtual AED before taking the examination [22]. Unlike our study, the participants had hands-on practice using an AED.

The positive findings regarding the learning outcomes of this study are promising, but they should be interpreted with caution due to the absence of a proper control group. Participants in the experimental group who used MFU BLiS VR were able to follow the BLS operation steps, analyze the situation, and make correct decisions for each scenario. Furthermore, they could replay the lessons as needed, allowing them to review and reinforce their knowledge and understanding, leading to BLS practice accuracy. With this, our study supported the notion that individuals who underwent BLS training under a virtual reality simulation environment gained a higher level of BLS knowledge and practical skills than traditional training. In a community-based study conducted in Spain, the researchers reported that immediately after completing the training course, the experimental group, which received BLS training with a VR program, demonstrated significantly higher knowledge compared to the control group, which received traditional role-play training. However, they did not find significant differences in knowledge at the six-month follow-up [18]. In this current study, we did not evaluate knowledge retention at a later time. Thus, whether employing VR technology for BLS training would enhance knowledge retention needs further investigation.

For BLS practical skills, our finding was consistent with a previous study that tested a similar intervention. In that study, the experimental group that received VR BLS training demonstrated significantly better overall BLS performance than the control group, which received web-based training. BLS performance was measured using the observation checklist [21] similar to our study. For effective BLS practical skills, it is generally accepted that chest compression skills, including compression rate and depth, are both important for CPR outcomes. In our study, we assessed the quality of chest compressions by observing whether the compression rate fell within the standard criterion (100–120 min^−1^) and whether a “green” light on the manikin indicated adequate compression depth (50 mm.). In contrast, a randomized noninferiority trial conducted in the Netherlands involved 381 adult attendees. It compared the quality of chest compressions, specifically compression rate and depth between a VR training group and a face-to-face training group [29]. This study objectively measured compression rates (min^−1^) and depths (mm.) using CPR-certified manikins, thus recording detailed descriptions of trainee performance. The results showed that the VR group demonstrated comparable chest compression rates but not chest compression depths. Differences in how details of the quality of compression are measured may contribute to these varying findings, warranting further investigation.

Finally, the hypothesis that the experimental group would have greater satisfaction with the BLS learning than the control group was not supported in our study. This finding is inconsistent with previous research on VR BLS training [18] and VR mechanical ventilation education programs [14]. The disparities in findings may be attributed to differences in measurement methods. In the latter study, learning satisfaction was assessed using a single-item, 10-point numeric rating scale [14], while our study employed a composite scale measured on a Likert-point scale. Moreover, some participants informally reported a feeling of headache, dizziness, or nausea. These are common symptoms of cybersickness [30]. Such symptoms may cause participants to feel uncomfortable.

## Conclusions

The results of this study demonstrated the effectiveness of the MFU BLiS VR training course for adult OHCA, which led to a reduction in no-flow time and an improvement in BLS knowledge and practice skills. This training course can be scaled up to benefit elementary students, high school students, and undergraduate students by enhancing their BLS knowledge and practical skills. Implementing such training can increase the rate of bystander CPR, as participants become more willing to offer assistance in the case of adult OHCA. We recommend conducting an extended replication study with a larger sample size, using a randomized controlled trial design, and objectively measuring the quality of chest compression using a sophisticated manikin. Furthermore, at the policy level, there should be consideration for the implementation of VR and blended learning approaches in BLS training courses at schools and universities.

### Limitations

Our study has no exceptions for study limitations. We identified the following limitations that could be improved in the future. Firstly, the MFU BLiS VR  is a training course that was offered for participants as an extracurricular activity alongside their regular educational program. Those who chose to participate may have already possessed a strong self-determination to learn these skills. Consequently, this selection bias may potentially threaten internal validity. Secondly, the usability of the proposed MFU BLiS VR may be limited by its compatibility. Currently, it is only available on smartphone devices with Android OS (version 8 and above) and requires access to the university internet connection through the student’s account. Thirdly, similar to other VR learning tools, the use of the MFU BLiS VR may be restricted by users with visual impairments and individuals prone to severe cybersickness. As a result, the generalizability of the findings to these groups in the general public is limited. Lastly, our study did not include a detailed objective assessment of chest compression quality, particularly compression depth. Future studies should consider evaluating chest compression depth in greater detail.

### Supplementary Information

**Additional file 1.**

## Data Availability

The datasets used and/or analyzed during the current study are available from the corresponding author on reasonable request.

## Abbreviations

AED: Automated external defibrillator
BLS: Basic life support
BKQ: BLS Knowledge Questionnaire
BSC: The BLS Skill Checklist
CCF: chest compression fraction
CPR: Cardiopulmonary resuscitation
MFU: Mae Fah Luang University
OHCA: Out-of-hospital cardiac arrest
ROSC: the return of spontaneous circulation
VR: Virtual reality
