# Supplementary material for: Development and evaluation of a virtual reality basic life support for undergraduate students in Thailand: a project by Mae Fah Luang University (MFU BLiS VR)
Source: BMC Med Educ. 2023 Oct 19;23:782. doi: 10.1186/s12909-023-04764-6 (PMC10588006; doi:10.1186/s12909-023-04764-6)
Supplement: Supplementary file 1 — Additional file 1. [file 12909_2023_4764_MOESM1_ESM.docx]

**Supplemental File 1**

Descriptions of the MFU VR BLiS for adult OHCA training course

| **Time (minute)** | **Objective** | **Activity** |
| --- | --- | --- |
| 15 | - Brief introduction about the BLS for adult OHCA course and baseline measurement. | - The participants were introduced to the BLS for adult OHCA course.  - Self-administered questionnaires were used to assess the participants’ BLS knowledge using the BKQ. |
| 8 | - To enhance knowledge and understanding of BLS. | - All participants were provided with a video clip about BLS which was available on the Mae Fah Luang University Learning Innovation Institute (MLii) website (<https://innovation-mlii.mfu.ac.th/?page_id=2583>)  (Fig.1) |
|  |  | 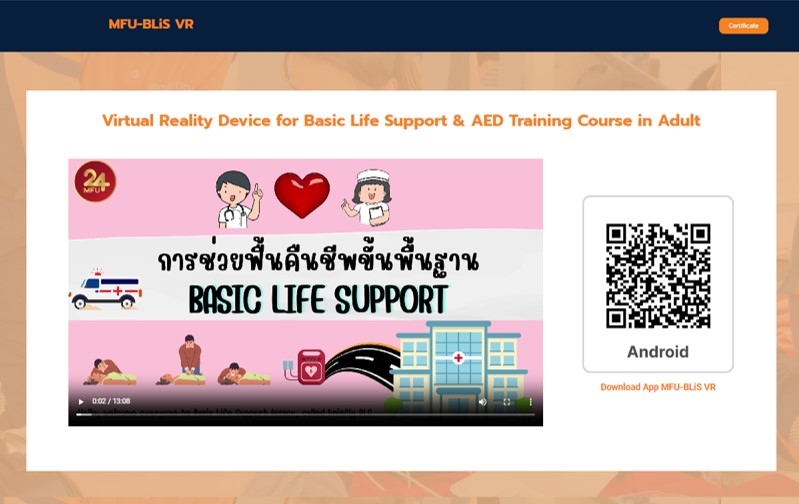  **Fig. 1** Display the webpage of MFU BLiS VR. |
| 15 | - To motivate the participants to learn and enhance knowledge, critical reasoning capacity, and confidence in BLS. | - The experimental group received the MFU VR BLiS (as shown in Fig. 2, Fig. 3) before practicing with a pillow.  - The control group received the MFU VR BLiS after the research process had ended. |
|  |  | 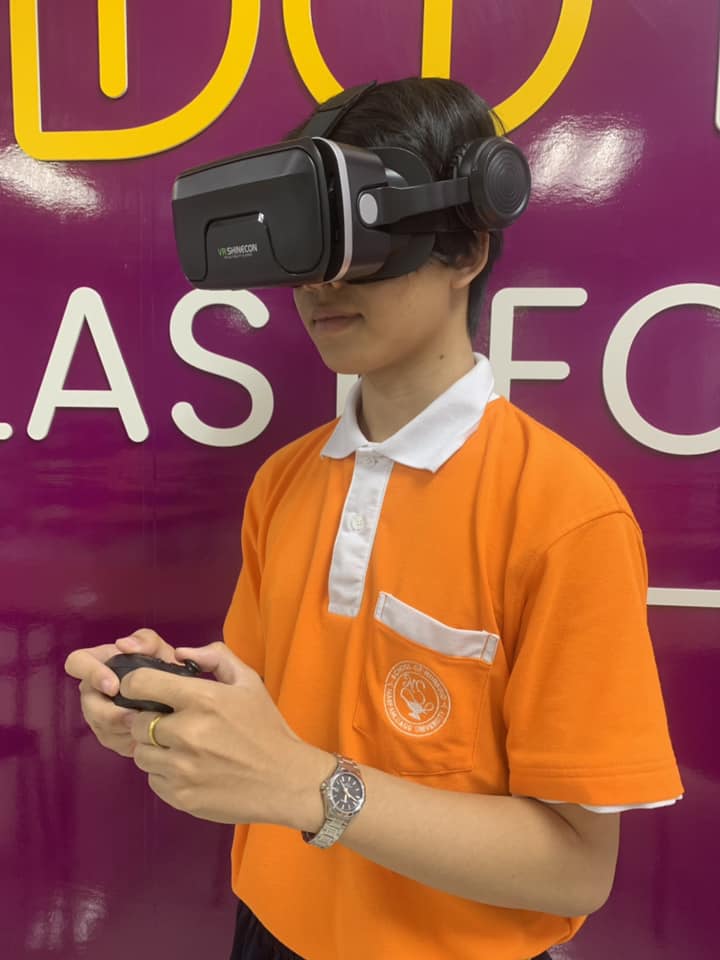  **Fig. 2** Display of a VR headset and VR remote controller. |
|  |  | 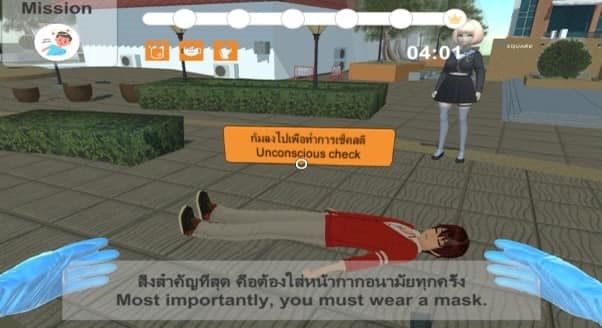  **Fig. 3** Participants viewed a VR BLS training with Thai voiceover and bilingual subtitles through a VR headset. |
| 15 | - To apply knowledge and enhance more frequent psychomotor practice skill of BLS, focusing on the participant’s position, rhythm, and steps for cardiopulmonary resuscitation (CPR). | - The participants are trained through self-administered CPR using a pillow, without the research assistant, at least once (Fig.4). |
|  |  | 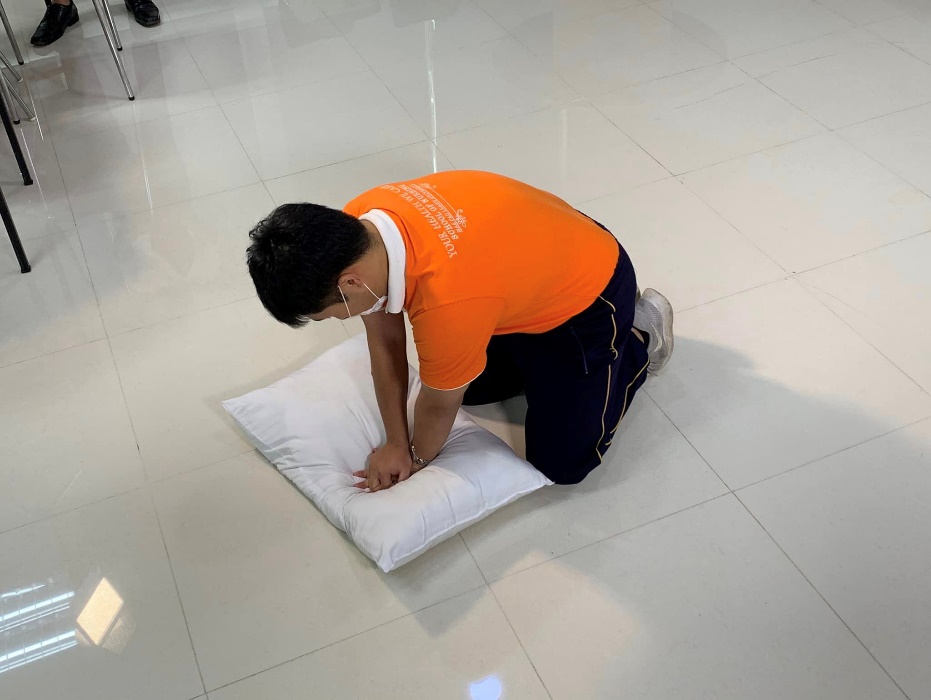  **Fig. 4** Practice BLS with a pillow, focusing on the participant’s position, rhythm, and steps of CPR. |
| 30 | - To practice BLS and use of an AED with a half-body training manikin.  - To assess the participants’ BLS skills. | - All participants received face-to-face training in standard one-person 30:2 CPR and practiced using the AED on a manikin in a classroom with the RA (Fig.5). The participants were instructed on how to use a face mask for assisted ventilation. However, hands-only (compression-only) bystander CPR was mentioned in the event of an unknown situation, especially during the COVID-19 pandemic.  - After practicing BLS skills, each participant was evaluated on CPR performance and capability of AED use by using the BSC. Correct CPR performance was defined in accordance with the 2020 AHA guidelines. |
|  |  | 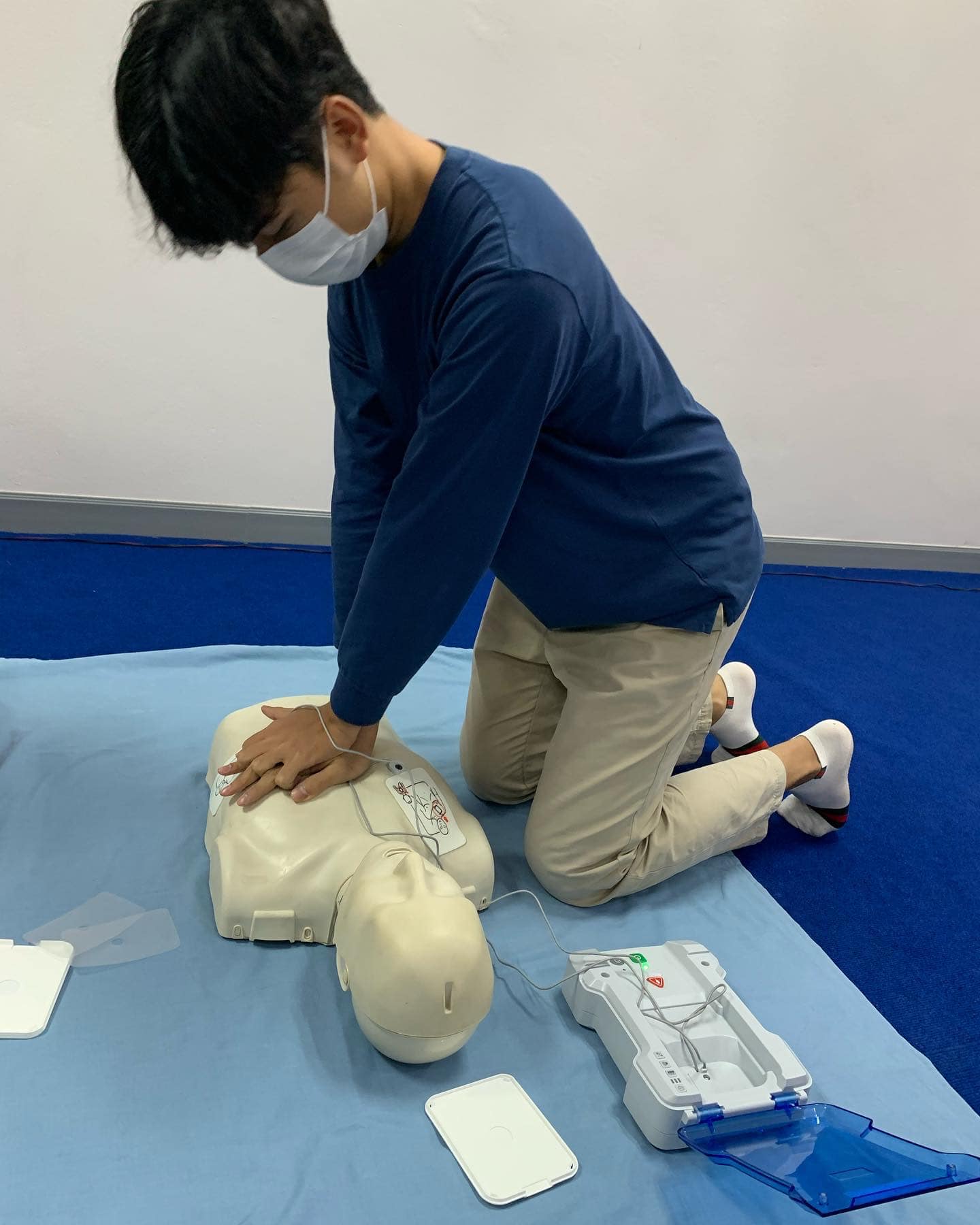  **Fig. 5** Practice BLS and use of an AED with a half-body training manikin in the classroom under the RA’s supervision. |
| 15 | - To assess post-test of knowledge on BLS. | - The participants’ knowledge of BLS was assessed by self-administering the BKQ. |
